# Validation of the Binge Eating Disorder Questionnaire (BED-Q) in Individuals with Type 2 Diabetes

**DOI:** 10.1186/s40337-026-01631-9

**Published:** 2026-05-21

**Authors:** Jakob Linnet, Nikolaj Vang Nielsen, Pernille Fiil Nybo, Erik Christiansen, Mia Beck Lichtenstein

**Affiliations:** 1 Clinic on Gambling- and Binge Eating Disorder, Occupational and Environmental Clinic, Odense University Hospital, Odense J. B. Winsløws Vej 4, Entrance 20, 15th Floor, Odense, Denmark; 2 Department of Clinical Research, Faculty of Health Sciences, University of Southern Denmark, Odense, Denmark; 3 Research Unit of Child and Adolescent Psychiatry, Odense University Hospital, Odense, Denmark; 4 Steno Diabetes Centre Odense, Odense University Hospital, Odense, Denmark; 5 Centre for Suicide Research, Odense, Denmark; 6 Research Unit for Psychiatry (Aabenraa), Hospital Sønderjylland, Department of Regional Health Research, University of Southern Denmark, Aabenraa, Denmark; 7 Department of Psychology, University of Southern Denmark, Odense, Denmark

**Keywords:** Binge Eating Disorder, Surveys and questionnaires, Psychometrics, Validity and reliability, Clinical assessment tools, Diagnostic screening programs

## Abstract

**Background:**

Binge Eating Disorder (BED) is an eating disorder characterized by recurrent episodes of binge eating accompanied by a sense of loss of control. This study aimed to examine the reliability, validity, and classification accuracy of a new instrument, the Binge Eating Disorder Questionnaire (BED-Q), in a sample of 364 individuals diagnosed with Type 2 diabetes (T2D).

**Methods:**

A total of 364 participants were included from a Danish cohort of 2,465 individuals with T2D. BED diagnosis was established using the Structured Clinical Interview for the DSM (SCID) based on the DSM-5 criteria, which served as the reference standard. The BED-Q was evaluated for internal consistency and unidimensionality using Cronbach’s alpha and confirmatory factor analysis (RMSEA, CFI, TLI, SRMR). Receiver Operating Characteristic (ROC) analyses assessed diagnostic performance, and optimal cut-off values were determined using the area under the curve (AUC), sensitivity, specificity, Positive Predictive Value (PPV), Negative Predictive Value, and Youden’s index.

**Results:**

The BED-Q showed excellent internal consistency (Cronbach’s α = 0.94; average inter-item correlation = 0.68), and confirmatory factor analysis supported a one-factor model with good final model fit (χ² (11) = 8.239, *p* = 0.692, CFI = 1.000, TLI = 1.006, RMSEA = 0.000, SRMR = 0.014). ROC analyses indicated good diagnostic accuracy (AUC = 0.86). A cut-off score of 12 provided optimal discrimination between BED and non-BED cases (Youden’s index = 0.71, sensitivity = 0.91, specificity = 0.81, PPV = 0.77, NPV = 0.92).

**Conclusions:**

The BED-Q demonstrated excellent reliability, unidimensionality, and diagnostic accuracy relative to a gold standard structured clinical interview. These findings support the use of the BED-Q as a valid screening instrument for BED. A cut-off score of 12 provided optimal sensitivity and specificity, consistent with DSM-5 and ICD-11 criteria. Further validation in diverse populations is recommended.

**Supplementary Information:**

The online version contains supplementary material available at 10.1186/s40337-026-01631-9.

## Abstract (plain language summary)

Binge Eating Disorder (BED) is an eating disorder characterized by eating large amounts of food while feeling a loss of control over one’s eating. This study examined whether a new questionnaire, the Binge Eating Disorder Questionnaire (BED-Q), can reliably identify people who may have BED. We tested the BED-Q in 364 adults with Type 2 diabetes from a large Danish health study. We looked at whether the questions measured the same underlying problem (binge eating), whether the scale was consistent, and how accurately it could distinguish between people with and without BED. The BED-Q performed very well on all tests, showing that it measures binge eating symptoms in a clear and reliable way. We also identified a total score of 12 or higher as the best cut-off point for indicating possible BED. At this level, the questionnaire correctly identified most people with BED and correctly excluded most people without the disorder. These results suggest that the BED-Q is a useful and accurate tool for screening BED. Further research should examine how well it works in the general population and other patient groups.

**Supplementary Information:**

The online version contains supplementary material available at 10.1186/s40337-026-01631-9.

## Introduction

Binge Eating Disorder (BED) is characterized in the DSM-5 by recurrent episodes of eating unusually large amounts of food accompanied by a sense of loss of control, occurring at least once a week for three months. These episodes are associated with marked distress and at least three behavioral indicators (such as eating rapidly, eating until uncomfortably full, eating when not hungry, eating alone due to embarrassment, or feeling disgusted or guilty afterward), without regular compensatory behaviors as seen in bulimia nervosa [1]. In the ICD-11, BED is similarly defined by recurrent binge-eating episodes involving loss of control and marked distress, but ICD-11 emphasizes clinical assessment of functional impairment or psychological distress, rather than behavioral thresholds, allowing for context-sensitive assessment across diverse cultural and clinical settings [2].

Lifetime prevalence of BED ranges from 0.8% to 1.9% in the WHO Mental Health Surveys, which is a higher prevalence than that of bulimia nervosa [3]. BED typically has an onset in late adolescence or early adulthood with a median onset around the age of 20 [4]. Approximately one-third of individuals with BED are men, while two-thirds are women [5, 6].

BED is frequently accompanied by a range of psychiatric and medical comorbidities that contribute to its clinical complexity and treatment challenges. More than three out of four BED patients have one or more comorbid psychiatric disorder in their lifetime [7–9]. Medically, BED is strongly associated with obesity-related conditions, including type 2 diabetes, high cholesterol, and hypertension [7, 8]. These psychiatric and metabolic comorbidities exacerbate distress and functional impairment and may complicate treatment response and long-term prognosis. Consequently, comprehensive assessment and integrated treatment approaches are essential to effectively address the multifaceted nature of BED.

The prevalence of eating disorders in patients with diabetes has previously been investigated and one study [10] found that 21.8% met the criteria for at least one DSM-5 eating disorder diagnosis. BED in patients with type 2 diabetes (T2D) has also been investigated and prevalence rates of 1.2-8.0% have been reported in a systematic review [11] which also found that BED was associated with higher weight. A study by Nicolau et al. [12] found a BED prevalence of 12.2% in patients with T2D which was also associated with higher weight. Thus, T2D patients constitute a population with increased risk of comorbid BED. Systematic screening for BED using valid instruments is recommended in high-risk populations, including people with overweight or diabetes [13].

Despite its prevalence, BED is often overlooked in health care settings [14]. Moreover, overweight patients are frequently subjected to stigmatization, including perceptions that they are lazy, ignorant, or personally responsible for their weight [15]. Such stigma contributes to mismanagement and may exacerbate the difficulties faced by individuals with BED.

The Binge Eating Disorder Questionnaire (BED-Q) was developed as a brief, user-friendly screening tool designed to quickly and reliably identify BED in health care settings. It can be administered in just a few minutes by general practitioners, nurses, psychologists, dietitians, and other health care professionals. The questionnaire is intended to be easily understood by patients while covering all diagnostic criteria necessary for establishing a diagnosis.

The BED-Q consists of nine items. Items 1–7 reflect the DSM-5 diagnostic criteria, item 8 assesses compensatory behaviors such as vomiting, and Item 9 measures distress associated with binge episodes. The questionnaire can also be adapted for use with ICD-11 diagnostic criteria. Evidence from a Danish validation of the EDE-Q [16] provides support for the construct validity of the BED-Q. Significant positive associations between the EDE-Q and the BED-Q were observed across four samples: (1) patients with anorexia nervosa, bulimia nervosa, or unspecified eating disorders (*n* = 101); (2) patients with BED (*n* = 300); (3) recreational athletes (*n* = 404); and (4) elite athletes (*n* = 526). These findings suggest that the BED-Q is a valid instrument for identifying binge eating symptoms in both clinical and non-clinical populations. Although previous studies have reported data on the BED-Q [16–20], this is the first validation study on the psychometric properties of the BED-Q.

The overall aim of the present study was to investigate the psychometric properties of the BED-Q in a sample of people with T2D. Compared with other measures, such as the Eating Disorder Examination Questionnaire (EDE-Q) [21] and the Binge Eating Scale (BES) [22], the BED-Q is concise and straightforward to score, yet provides a more nuanced assessment of BED symptoms than, e.g., the Binge Eating Disorder Screener-7 (BEDS-7) [23].

In addition to evaluating internal consistency and factor structure, the present study examined the diagnostic accuracy of the BED-Q by assessing its ability to differentiate between individuals with and without BED, using the Structured Clinical Interview for DSM (SCID) [24], based on DSM-5 criteria, as the reference standard.

We hypothesized that the BED-Q would demonstrate high reliability (as indicated by Cronbach’s alpha and confirmatory factor analysis) and strong diagnostic performance, reflected in high sensitivity and specificity in identifying BED cases.

## Methods

### Participants and procedures

 Participants were recruited from two sources. First, 537 individuals were selected from the Danish Centre for Strategic Research in Type 2 Diabetes (DD2) cohort (n = 2,465) [25]and invited for telephone assessment as part of a Ph.D. project on BED and type 2 diabetes. Second, an additional 43 individuals were recruited from Syddansk Overvægts Initiativ (SDOI; the South Danish Obesity Initiative) at the Clinic for Nutrition, Odense University Hospital, and from other diabetes clinics in the Region of Southern Denmark, and were assessed in person. Both groups were assessed using the BED-Q and a modified eating disorders module of the Structured Clinical Interview for DSM (SCID-I) [24] adapted to the DSM-5 diagnostic criteria, covering anorexia nervosa, bulimia nervosa, and BED.

SCID-I diagnoses were assessed either over the phone or in person, while the BED-Q and a patient information questionnaire were distributed to patients in the DD2 cohort using a REDCap database, supported by the Odense Patient Data Explorative Network (OPEN) at the Region of Southern Denmark, the University of Southern Denmark, and Odense University Hospital (OUH). OPEN complies with Danish national data protection regulations and the General Data Protection Regulation (GDPR), ensuring secure data management and processing.

Participants were included in the study if they completed both the BED-Q and the SCID-I. Of the 537 invited from the DD2 cohort, 321 completed the SCID-I over the phone and the remaining 216 were excluded due to non-response, declining to participate, or missing data. All 43 in-person participants completed assessment. The final analytic sample comprised 364 participants (321 telephone + 43 in person), drawn from a combined invited sample of 580.

Based on the SCID-I assessment, participants were classified as either meeting criteria for BED (BED+) or not meeting criteria for BED (BED−). Descriptive demographic and clinical characteristics for these groups are presented in Table 1.

Interviewers were trained and supervised by the first author (JL), a clinical psychologist and specialist in psychotherapy. Interviewers were blinded to the BED-Q response at the time of interviewing. Blinding was subsequently removed during data analysis, which involved both the first and second authors (JL and NVN).

### Binge eating disorder questionnaire (BED-Q)

The BED-Q is a 9-item questionnaire. Items 1–7 constitute the total score (range 0–35) and correspond to the DSM-5 diagnostic criteria: (A1) eating larger amounts of food within a short time (two hours); (A2) losing control over one’s eating; (B1) eating faster than normal; (B2) eating until uncomfortably full; (B3) eating without being hungry; (B4) eating alone; and (B5) experiencing negative feelings after overeating.

Items 1–7 are rated as follows: “No” (0), “Yes, less than once a week” (1); “Yes, 1–3 times a week” (2); “Yes, 4–7 times a week” (3); “Yes, 8–13 times a week” (4); “Yes, 14 or more times a week” (5). The total score is interpreted as follows: 0 = no symptoms; 1–9 = subclinical symptoms of BED; 10–14 = mild BED; 15–21 = moderate BED; 22–28 = severe BED; 29–35 = extremely severe BED.

Item 8 assesses compensatory behaviors such as self-induced vomiting, while item 9 assesses whether binges are experienced as distressing. The BED-Q is freely available and is included as Supplementary Material.

### Structured clinical interview for the DSM (SCID)

The Structured Clinical Interview for DSM-IV (SCID-I) [24] is a clinical interview assessing the most common types of psychopathology using the DSM criteria. We used a modified SCID-I version adapted to the DSM-5 diagnostic criteria. Participants were assessed on anorexia nervosa, bulimia nervosa and BED.

### Age, gender and BMI measures

Age and gender were determined from the Danish personal identification number (similar to a Social Security number), while BMI was calculated based on self-reported measures provided during assessment.

### Statistical analysis

All statistical analyses were conducted using Stata version 18.0 SE (StataCorp, College Station, TX, USA). Descriptive analyses were performed to summarize participant characteristics and item responses. Independent samples t-tests and chi-square analyses were used to examine differences in age, BMI, and gender distribution between participants with BED and those without.

Internal consistency of the seven Likert-scale items of the BED-Q (items 1–7) was assessed using Cronbach’s alpha. Unidimensionality was evaluated using the Scale Reliability Coefficient and the Average Inter-item Correlation. For each item, we assessed the fit to a normal distribution as well as multivariate normality using Mardia’s, Henze-Zirkler’s, and Doornik-Hansen’s tests. Results indicated a significant violation of normality assumptions. Therefore, we employed maximum likelihood estimation with the Satorra-Bentler correction to obtain robust standard errors and fit indices. Missing data on the BED-Q indicators were handled via listwise deletion, resulting in a complete-case analysis.

Confirmatory factor analysis (CFA) was performed to assess model fit. To identify areas of localized strain, we inspected modification indices derived from standard Maximum Likelihood estimation. Adjustments were evaluated in descending order of magnitude and applied to the Satorra-Bentler corrected model only if they were theoretically justifiable and resulted in stable parameter estimates. This iterative process yielded a final model with three correlated error terms, at which point further modifications provided negligible improvement to model parsimony. Model fit was assessed using the root-mean-square error of approximation (RMSEA), comparative fit index (CFI), Tucker–Lewis index (TLI), and standardized root-mean-square residual (SRMR).

To examine the association between BED-Q scores and diagnostic status, we conducted a logistic regression with BED as the dependent variable. The model was used to estimate predicted probabilities of BED across the full range of BED-Q scores, providing a visual representation of how the likelihood of diagnosis increased with higher scores. This analysis was conducted to support the subsequent cut-off determination based on sensitivity, specificity, and Youden’s index.

Finally, we conducted Receiver Operating Characteristic (ROC) curve analyses to evaluate the performance of the BED-Q as a binary classifier for BED diagnosis and calculated the Area Under the Curve (AUC) to evaluate the sensitivity and specificity of the scale. We used Youden’s Index (J) to identify an optimal cut-off point for each possible total score on the scale (range 0–35). We also calculated Positive Predictive Value (PPV) and Negative Predictive Value (NPV) to evaluate the performance of the scale at the optimal cut-off.

## Results

**Fig. 1:**
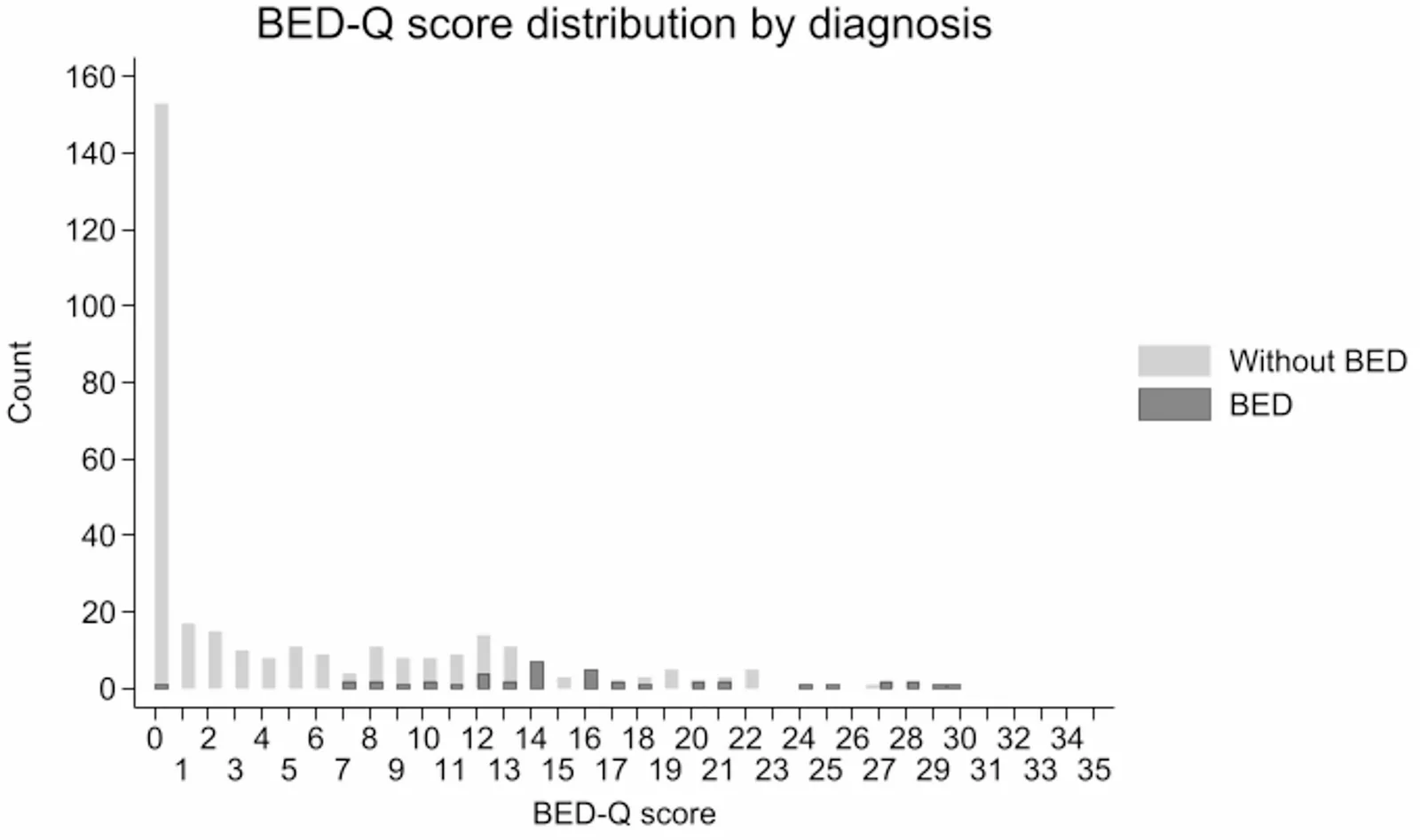
BED-Q score distribution by diagnosis

Table 1 shows demographic and clinical characteristics across participants with and without BED. Participants with BED were significantly younger and had significantly higher BMI than participants without BED. No significant differences in gender distribution were seen between the two groups. Participants with BED scored significantly higher on all seven BED-Q items. Figure 1 shows the BED-Q score distribution by diagnosis.

**Table 1:** Participant characteristics and BED-Q distribution according to BED status

Characteristics	Total (*n* = 364)	BED+ (*n* = 42)	BED− (*n* = 322)	*p*-value<
Age, mean (SD)	57.61 (8.45)	52.45 (9.94)	58.28 (8.02)	0.0006
Female, n (%)	221 (60.71%)	31 (73.81%)	190 (59.01%)	0.065
BMI, mean (SD), n*	33.08 (8.37), 254	43.07 (10.54), 31	31.69 (6.99), 223	0.0001
BED-Q score, mean (SD)	6.02 (7.33)	16.05 (6.86)	4.71 (6.32)	0.0001
1a: Binge, mean (SD)	1.17 (1.39)	2.71 (1.13)	0.97 (1.3)	0.0001
1b: Control, mean (SD)	1.07 (1.37)	2.62 (1.08)	0.87 (1.27)	0.0001
2a: Faster, mean (SD)	0.76 (1.20)	2.12 (1.49)	0.58 (1.04)	0.0001
2b: Uncomfortable, mean (SD)	0.88 (1.20)	2.21 (1.20)	0.71 (1.08)	0.0001
2c: Hungry, mean (SD)	0.87 (1.21)	2.24 (1.14)	0.69 (1.10)	0.0001
2d: Embarrassment, mean (SD)	0.52 (1.02)	1.95 (1.32)	0.33 (0.81)	0.0001
2e: Guilty, mean (SD)	0.76 (1.17)	2.19 (1.17)	0.57 (1.03)	0.0001

BMI data were missing for some participants. BED+ denotes participants meeting DSM-5 criteria for BED based on the SCID interview; BED− denotes participants not meeting these criteria.

BED-Q total scores in the sample ranged from 0 to 30 (see Fig. 1). This indicates that participants spanned the full range of the scale from no symptoms to severe levels of binge-eating pathology, although scores in the extreme range (29–35) were infrequent.

Table 2 shows the inter-item correlation for the seven Likert-scale BED-Q items (items 1–7).

**Table 2:** Inter-item correlation matrix

	1a: Binging	1b: Loss of control	2a: Eating Faster	2b: Uncom-fortably full	2c: Eating, without hunger	2d: Eating alone	2e: feeling guilty afterwards
1a: Binging	1						
1b: Loss of control	0.9272	1					
2a: Eating faster	0.6971	0.6707	1				
2b: Uncomfortably full	0.7123	0.7571	0.6662	1			
2c: Eating without hunger	0.8218	0.8227	0.6961	0.7557	1		
2d: Eating alone	0.5915	0.5909	0.5346	0.5218	0.5662	1	
2e: Feeling guilty afterwards	0.6834	0.6725	0.5621	0.6487	0.7191	0.6344	1

The Cronbach’s alpha coefficient was 0.94 and the Average Inter-item Correlation was 0.6787 [0.52, 0.93]. A normal distribution analysis showed a positive skew and a wide (platykurtic) distribution of the data due to more participants with lower BED-Q scores.

**Fig. 2:**
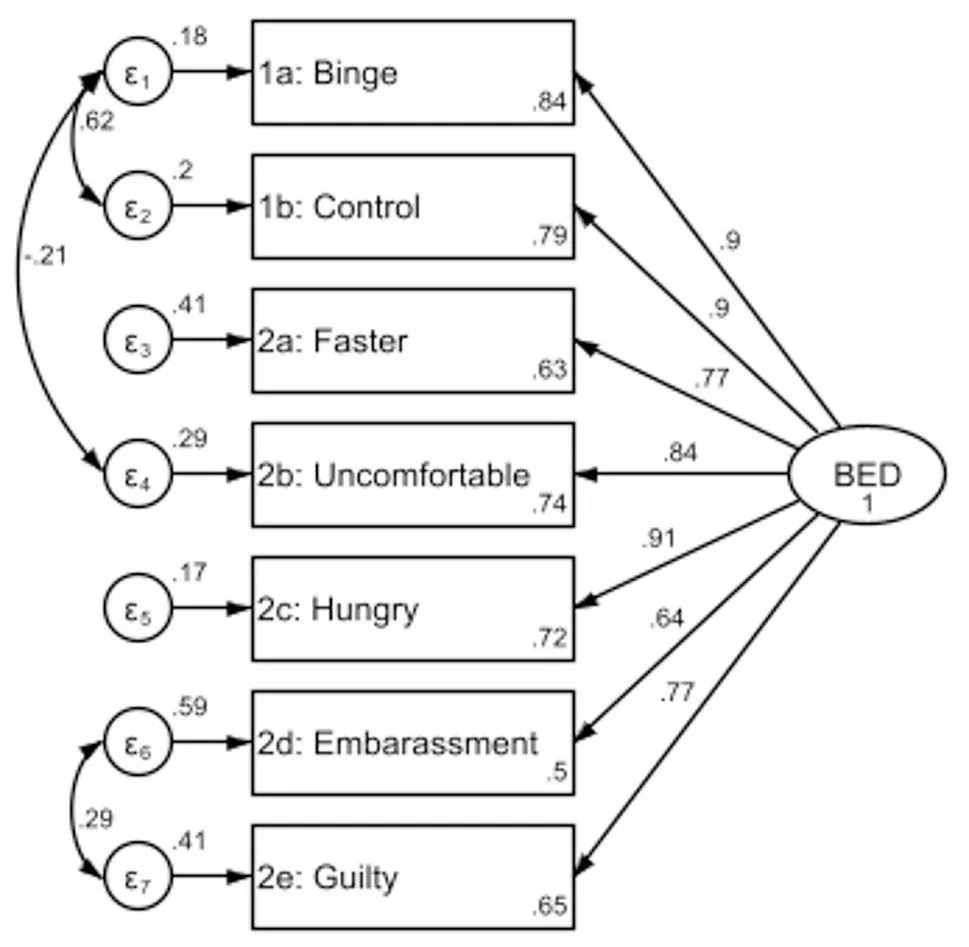
Measurement model of the BED-Q with one latent BED factor

The confirmatory factor analysis used a stepwise refinement process, beginning with a simple model and subsequently adjusting based on modification indices to improve model fit. The initial model showed poor fit (χ² [14] = 53.615, *p* < 0.001, CFI = 0.954, TLI = 0.931, RMSEA = 0.088, SRMR = 0.044). Allowing for covariance between items A1 (Binge eating) and A2 (Loss of control) improved the fit (χ² [13] = 23.494, *p* = 0.036, CFI = 0.988, TLI = 0.980, RMSEA = 0.047, SRMR = 0.027). Further adjustments for covariance between items B4 (eating alone due to feeling embarrassed) and B5 (feeling guilty after overeating) yielded additional improvement (χ² [12] = 14.197, *p* = 0.288, CFI = 0.997, TLI = 0.996, RMSEA = 0.022, SRMR = 0.015). A final adjustment for a smaller negative covariance between items A1 (Binge eating) and B3 (eating to uncomfortably full) resulted in a satisfactory model fit (χ² [11] = 8.239, *p* = 0.692, CFI = 1.000, TLI = 1.006, RMSEA = 0.000, SRMR = 0.014), supporting a one-factor model with a single latent factor as the best fit for the data (see Table 3; Fig. 2). Theoretical interpretations of the covariations are discussed below.

**Table 3:** Model fit indices, internal consistency, and diagnostic accuracy for the unadjusted and adjusted BED-Q models using Maximum Likelihood estimation with Satorra-Bentler corrections

Model	Χ^2^ (df, *p*)	RMSEA [90% CI]	CFI	TLI	SRMR	Cronbach’s α	AUC [95% CI]
CFA: 1 factor, no correlated uniqueness	56.615 (14, p < 0.0001)	0.088 [0.064, 0.114]	0.954	0.931	0.044	0.9374	0.8799 [0.83, 0.93]
CFA: 1 factor, 3 correlated uniquenesses	8.239 (11, p = 0.692)	0.000 [0.000, 0.043]	1.000	1.006	0.014		

**Fig. 3:**
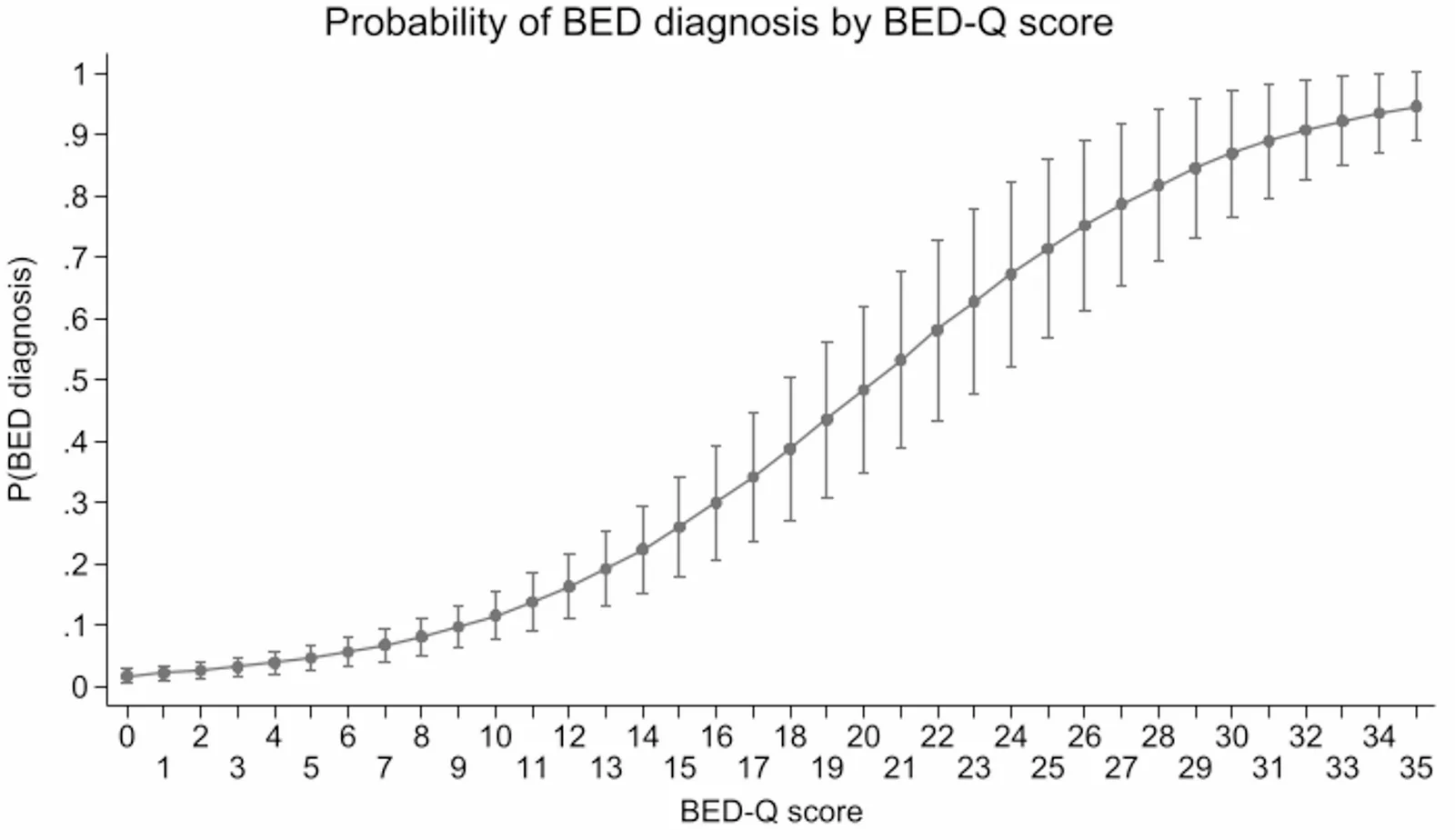
Predicted probability of binge eating disorder (BED) diagnosis as a function of BED-Q total score, estimated by logistic regression, with 95% confidence intervals

The logistic regression model showed a clear positive association between total BED-Q score and the probability of meeting diagnostic criteria for BED (see Fig. 3). The predicted probability increased steadily across the score range, indicating that higher BED-Q scores were consistently associated with greater likelihood of BED diagnosis. This pattern supports the scale’s criterion validity.

**Fig. 4:**
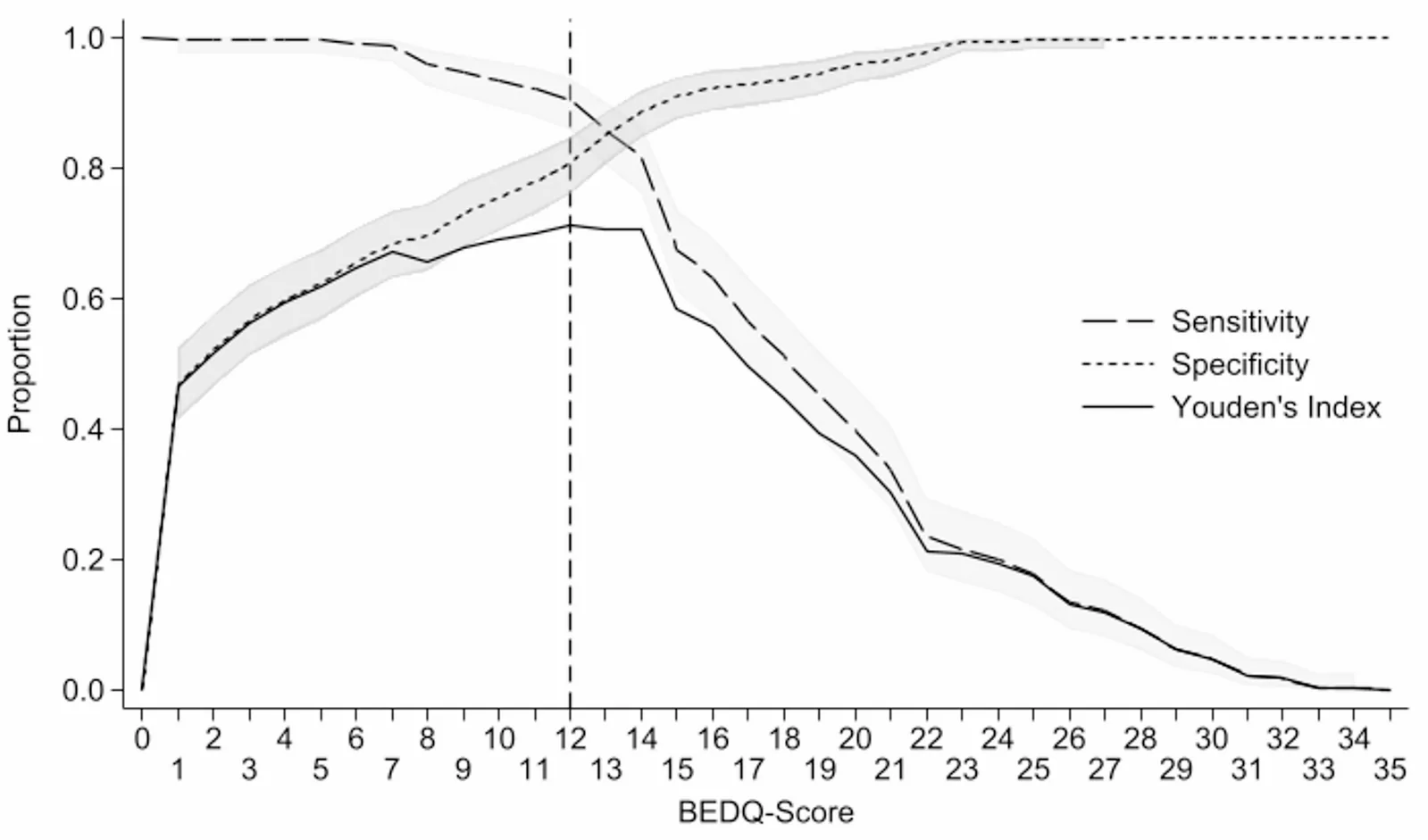
Cut-off analysis plot

Finally, the cut-off analysis showed that a total score of 12 represented the optimal threshold for distinguishing between participants with and without BED (see Fig. 4). This cut-off corresponded to a Youden’s index of 0.71, sensitivity of 0.91, specificity of 0.81, PPV of 0.77, NPV of 0.92, and an overall ROC value of 0.86 (see Table 4).

**Table 4:** Diagnostic performance of the BED-Q around the optimal cut-off

Cutoff	Sensitivity	Specificity	J	PPV	NPV
8	0.961	0.696	0.657	0.692	0.962
9	0.948	0.730	0.678	0.713	0.952
10	0.935	0.755	0.690	0.730	0.943
11	0.922	0.779	0.701	0.747	0.934
12	0.905	0.807	0.712	0.768	0.923
13	0.857	0.850	0.707	0.802	0.894
14	0.818	0.887	0.705	0.836	0.873
15	0.675	0.911	0.586	0.843	0.798
16	0.632	0.923	0.555	0.854	0.780

## Discussion

Our findings indicate that the BED-Q functions as a unidimensional scale with strong psychometric properties, including satisfactory internal consistency, construct validity, and diagnostic accuracy. The confirmatory factor analysis supported a single-factor structure reflecting the seven DSM-5 diagnostic criteria. The internal consistency was excellent, with a Cronbach’s alpha of 0.94, consistent with Nunnally’s [26] recommendations for clinical measures. Together, these results suggest that the BED-Q provides a reliable and valid tool for screening for BED in both research and clinical contexts.

The BED-Q differs from other brief eating disorder screening tools such as SCOFF [27] and BEDS-7 [23] because it corresponds with the diagnostic criteria for BED in both DSM and ICD and it can assess the level of BED severity. SCOFF has been validated in young Danes [28] and showed good psychometric properties. However, SCOFF was originally developed to assess anorexia and bulimia, not BED [27]. BEDS-7 has been validated in 42 countries [29] but never in a Danish population and the original validation study showed low specificity of 38.7% [23]. BEDS-7 fails to assess “eating faster than normally” and “eating until uncomfortably full” and this may explain the low specificity.

One scale has been developed to screen for disordered eating in patients with diabetes, the Diabetes Eating Problem Survey – Revised (DEPS-R), but it was not specifically developed for BED [10] and the scale showed a three-factor structure.

The cut-off analysis identified a BED-Q score of 12 as the optimal threshold for distinguishing individuals with probable BED from those without, reflecting a strong overall classification performance (AUC = 0.86). At this threshold, the scale demonstrated high sensitivity (0.91) and specificity (0.81), indicating that most individuals with BED were correctly identified, while false positives were relatively limited. The positive predictive value of 0.77 suggests that individuals scoring 12 or above have a high likelihood of meeting criteria for BED, whereas the negative predictive value of 0.92 indicates that those scoring below 12 are unlikely to meet criteria for BED. These results highlight the clinical utility of the cut-off score and support the BED-Q as a useful screening tool for identifying individuals at elevated risk of BED.

While the cut-off score of 12 provides satisfactory classification accuracy for identifying probable BED, the BED-Q should be used in conjunction with clinical evaluation whenever possible. Clinical observations indicate that some individuals who experience loss of control over eating and clinically significant distress (consistent with ICD-11 criteria) may not reach a score of 12 or higher, as the BED-Q cut-off was calibrated to reflect DSM-5 diagnostic requirements. This discrepancy underscores the importance of clinical judgment when interpreting BED-Q scores and highlights that screening tools cannot fully replace diagnostic assessment.

Although the Average Inter-item Correlation of 0.6787 exceeds the upper limit of 0.50 recommended by Clark and Watson [30], this result is consistent with the nature of the BED-Q as a measure of a narrow, specific clinical syndrome. The high convergence suggests that the items capture a tightly clustered set of symptoms rather than a broad, multi-faceted construct.

Our data showed a high covariation between eating larger amounts of food (A1) and feeling a loss of control (A2). Clinically, many patients are unsure what constitutes a “larger amount than most people would eat under similar circumstances”. Many describe their binge eating episodes as “eating more than intended” and a feeling of “losing control” over their eating, both of which may reflect a loss of control: “Eating more than intended” may reflect a behavioral loss of control and the feeling of losing control may reflect an emotional dimension of control loss. This could explain the high inter-correlation in our findings between “eating larger amounts” (A1) and “feeling of losing control” (A2) on the BED-Q.

Since it is clinically relevant to maintain both item A1 and A2 in the BED-Q to differentiate between portion size and loss of control, clinicians might have to take extra care to help patients distinguish between “larger amounts of food” and “feelings of loss of control”, respectively. In the DSM-5 both symptoms must be present to meet criteria for a binge eating episode. The ICD-11, however, distinguishes between “objective” binge eating, where both symptoms are present, and “subjective” binge eating which is characterized by “eating amounts of food that might be objectively considered to be within normal limits but are subjectively experienced as large by the individual” [2].

Subjective binge eating is relevant to assess in all BED screening, but is particularly important among patients with increased satiation, post-bariatric surgery, or undergoing weight loss treatments such as GLP-1 receptor agonists [31]. Subjective binge eating episodes may involve smaller quantities of food but still be clinically significant if associated with feelings of loss of control. Patients with subjective binge eating may not meet the DSM-5 criteria for BED, which emphasizes objective binge episodes, but may fully meet diagnostic criteria for BED under the ICD-11, which recognizes subjective binge eating. The BED-Q is a valuable screening tool in this context, as it captures both objective and subjective binge eating episodes, supporting the identification of BED according to both DSM-5 and ICD-11 criteria.

Eating alone due to feeling embarrassed about binge eating (B4) and feeling disgusted, depressed or guilty afterwards (B5) also showed a high covariation on the BED-Q. Shame and guilt may be another distinction, which is difficult to make for some BED patients. Shame and guilt are conceptually distinct: Shame involves a negative evaluation of the global self (“*I* did that horrible thing”), while guilt involves a negative evaluation of a specific behavior (“I *did* that horrible *thing*”) [32]. However, many people with BED report feeling shame not only when binge eating alone, but also afterwards, which is often associated with self-critical thinking of being a weak or inept person. These self-critical thoughts may reinforce or exacerbate the binge eating behavior and trigger feelings of helplessness or hopelessness about changing the eating behavior, which can take on a self-harming or punishing character (“I’m no better, so I don’t deserve better”). A study of binge eating in bulimia nervosa found that binge eating was uniquely related with shame, separate from guilt [33].

While DSM-5 theoretically distinguishes between eating alone due to shame (“*I* ate too much, again!”) and feeling guilty over the behavior afterwards (“I *ate* too much, again!”) the empirical evidence suggests that shame rather than guilt is involved both in eating alone due to feeling embarrassed and feeling shame over the behavior afterwards. Therefore, it is possible that shame is the underlying construct for B4 (eating alone due to shame) and B5 (feeling guilty afterwards), thus accounting for the inter-correlation of items B4 and B5.

This study has several limitations. First, the sample consisted exclusively of individuals with type 2 diabetes, which may limit the generalizability of the findings to broader clinical or community populations. Replication in non-diabetic samples is therefore necessary to determine whether the BED-Q demonstrates similar psychometric properties across diverse clinical contexts. Second, participants with BED in the current study were significantly younger and had higher BMI compared to those without BED. These group differences raise the possibility that age or BMI may have influenced item endorsement or diagnostic classification, and future studies should aim to replicate the findings in samples matched on key demographic and clinical variables. Third, the observed BED-Q scores in this study ranged from 0 to 30, spanning the full clinical range of the scale (no symptoms through extremely severe), although no participant reached the upper extreme-severity tail (29–35). Results should therefore be interpreted with caution for scores from 29 to 35. Finally, because binge-eating behaviors and their interpretation may vary across cultural contexts, evaluating the BED-Q in different languages and cultural settings is essential to ensure cross-cultural validity and clinical applicability.

## Conclusion

The BED-Q demonstrated strong internal consistency, unidimensionality, and good diagnostic accuracy, supporting its use as a reliable and valid screening tool for BED. A cut-off score of 12 showed optimal sensitivity and specificity for identifying probable BED cases, aligning closely with DSM-5 diagnostic criteria while also accommodating ICD-11’s inclusion of subjective binge eating. Further validation of the BED-Q in broader and cross-cultural populations is warranted.

## Supplementary Information

Below is the link to the electronic supplementary material.

Supplementary Material 1.

## Data Availability

The data that support the findings of this research are available from the corresponding author upon reasonable request.

## References

[1] American Psychiatric Association. Diagnostic and Statistical Manual of Mental Disorders. 5th ed., text rev. Washington, DC: American Psychiatric Publishing; 2022.

[2] World Health Organization [ICD-11]. International classification of diseases for mortality and morbidity statistics (11th Revision). Retrieved from https://icd.who.int/browse11/l-m/en. Geneva: WHO; 2018.

[3] Kessler RC, Berglund PA, Chiu WT, Deitz AC, Hudson JI, Shahly V, et al. The prevalence and correlates of binge eating disorder in the World Health Organization World Mental Health Surveys. Biol Psychiatry. 2013;73(9):904–14. 23290497 10.1016/j.biopsych.2012.11.020 PMC3628997

[4] Solmi M, Radua J, Olivola M, Croce E, Soardo L, Salazar de Pablo G, et al. Age at onset of mental disorders worldwide: large-scale meta-analysis of 192 epidemiological studies. Mol Psychiatry. 2022;27(1):281–95. 34079068 10.1038/s41380-021-01161-7 PMC8960395

[5] Lee-Winn AE, Reinblatt SP, Mojtabai R, Mendelson T. Gender and racial/ethnic differences in binge eating symptoms in a nationally representative sample of adolescents in the United States. Eat Behav. 2016;22:27–33. 27085166 10.1016/j.eatbeh.2016.03.021 PMC4983227

[6] Thornton LM, Watson HJ, Jangmo A, Welch E, Wiklund C, von Hausswolff-Juhlin Y, et al. Binge-eating disorder in the Swedish national registers: Somatic comorbidity. Int J Eat Disord. 2017;50(1):58–65. 27642179 10.1002/eat.22624 PMC5215312

[7] Hudson JI, Hiripi E, Pope HG Jr., Kessler RC. The prevalence and correlates of eating disorders in the National Comorbidity Survey Replication. Biol Psychiatry. 2007;61(3):348–58. 16815322 10.1016/j.biopsych.2006.03.040 PMC1892232

[8] Udo T, Grilo CM. Psychiatric and medical correlates of DSM-5 eating disorders in a nationally representative sample of adults in the United States. Int J Eat Disord. 2019;52(1):42–50. 30756422 10.1002/eat.23004

[9] Javaras KN, Pope HG, Lalonde JK, Roberts JL, Nillni YI, Laird NM, et al. Co-occurrence of binge eating disorder with psychiatric and medical disorders. J Clin Psychiatry. 2008;69(2):266–73. 18348600 10.4088/jcp.v69n0213

[10] Pinna F, Diana E, Sanna L, Deiana V, Manchia M, Nicotra E, et al. Assessment of eating disorders with the diabetes eating problems survey - revised (DEPS-R) in a representative sample of insulin-treated diabetic patients: a validation study in Italy. BMC Psychiatry. 2017;17(1):262. 28724422 10.1186/s12888-017-1434-8 PMC5518128

[11] Abbott S, Dindol N, Tahrani AA, Piya MK. Binge eating disorder and night eating syndrome in adults with type 2 diabetes: a systematic review. J Eat Disord. 2018;6:36. 30410761 10.1186/s40337-018-0223-1 PMC6219003

[12] Nicolau J, Simo R, Sanchis P, Ayala L, Fortuny R, Zubillaga I, et al. Eating disorders are frequent among type 2 diabetic patients and are associated with worse metabolic and psychological outcomes: results from a cross-sectional study in primary and secondary care settings. Acta Diabetol. 2015;52(6):1037–44. 25841588 10.1007/s00592-015-0742-z

[13] Harris SR, Carrillo M, Fujioka K. Binge-Eating Disorder and Type 2 Diabetes: A Review. Endocr Pract. 2021;27(2):158–64. 33554873 10.1016/j.eprac.2020.10.005

[14] White MA, Gianini LM. Binge Eating Disorder and Obesity. In: Alexander J, Goldschmidt AB, Grange DL, editors. A Clinician’s Guide to Binge Eating Disorder. N. Y.: Routledge; 2013.

[15] Diedrichs PC, Pihl RO. Weight Bias: Prejudice and Discrimination toward overweight and Obese People. In: Sibley CG, Barlow. K, editors. The Cambridge Handbook of the Psychology of Prejudice. Cambridge, UK: Cambridge University Press; 2017.

[16] Lichtenstein MB, Haastrup L, Johansen KK, Bindzus JB, Larsen PV, Støving RK et al. Validation of the Eating Disorder Examination Questionnaire in Danish Eating Disorder Patients and Athletes. J Clin Med. 2021;10(17). 10.3390/jcm10173976 PMC8432050 34501422

[17] Jensen ES, Linnet J, Holmberg TT, Tarp K, Nielsen JH, Lichtenstein MB. Effectiveness of internet-based guided self-help for binge-eating disorder and characteristics of completers versus noncompleters. Int J Eat Disord. 2020;53(12):2026–31. 32918321 10.1002/eat.23384

[18] Runge E, Jensen EK, Mathiasen K, Larsen PV, Hertz SPT, Holmberg TT, et al. Early development of treatment motivation predicts adherence and symptom reduction in an internet-based guided self-help program for binge eating disorder. Front Psychiatry. 2022;13:969338. 36276339 10.3389/fpsyt.2022.969338 PMC9583526

[19] Linnet J, Hertz SPT, Jensen ES, Runge E, Tarp KHH, Holmberg TT, et al. Days between sessions predict attrition in text-based internet intervention of Binge Eating Disorder. Internet Interv. 2023;31:100607. 36819741 10.1016/j.invent.2023.100607 PMC9930145

[20] Linnet J, Jensen ES, Runge E, Hansen MB, Hertz SPT, Mathiasen K, et al. Text based internet intervention of Binge Eating Disorder (BED): Words per message is associated with treatment adherence. Internet Interv. 2022;28:100538. 35480237 10.1016/j.invent.2022.100538 PMC9035730

[21] Fairburn CG, Beglin SJ. Assessment of eating disorders: interview or self-report questionnaire? Int J Eat Disord. 1994;16(4):363–70. 7866415

[22] Gormally J, Black S, Daston S, Rardin D. The assessment of binge eating severity among obese persons. Addict Behav. 1982;7(1):47–55. 7080884 10.1016/0306-4603(82)90024-7

[23] Herman BK, Deal LS, DiBenedetti DB, Nelson L, Fehnel SE, Brown TM. Development of the 7-Item Binge-Eating Disorder Screener (BEDS-7). Prim Care Companion CNS Disord. 2016;18(2). 10.4088/PCC.15m01896 PMC4956427 27486542

[24] First M, Spitzer R, Gibbon M, Williams J. Structured clinical interview for the DSM-IV Axix I disorders (SCID I/P. Version 2.0). New York: Biometrics Research Department, New York State Psychiatric Institute;; 1995.

[25] Type 2 Diabetes and Binge Eating Disorder (BED) (NCT06325670) [Internet]. U.S. National Library of Medicine. 2025. Available from: https://clinicaltrials.gov/ct2/show/NCT06325670

[26] Nunnally JC, Bernstein IH. Psychometric theory. 3rd ed. New York: McGraw-Hill; 1994.

[27] Morgan JF, Reid F, Lacey JH. The SCOFF questionnaire: assessment of a new screening tool for eating disorders. BMJ. 1999;319(7223):1467–8. 10582927 10.1136/bmj.319.7223.1467 PMC28290

[28] Lichtenstein MB, Hemmingsen SD, Stoving RK. Identification of eating disorder symptoms in Danish adolescents with the SCOFF questionnaire. Nord J Psychiatry. 2017;71(5):340–7. 28290749 10.1080/08039488.2017.1300322

[29] Gewirtz-Meydan A, Spivak-Lavi Z, Kraus SW, Nagy L, Koós M, Demetrovics Z, et al. Cross-Cultural Validation of the Binge Eating Disorder Screener-7 (BEDS-7) Across 42 Countries. Int J Eat Disord. 2025;58(5):926–38. 40040591 10.1002/eat.24365 PMC12067514

[30] Clark LA, Watson D. Constructing validity: Basic issues in objective scale development. Psychol Assess. 1995;7(3):309–19.

[31] Friedrichsen M, Breitschaft A, Tadayon S, Wizert A, Skovgaard D. The effect of semaglutide 2.4 mg once weekly on energy intake, appetite, control of eating, and gastric emptying in adults with obesity. Diabetes Obes Metab. 2021;23(3):754–62. 33269530 10.1111/dom.14280 PMC7898914

[32] Tangney JP, Stuewig J, Mashek DJ. Moral emotions and moral behavior. Annu Rev Psychol. 2007;58:345–72. 16953797 10.1146/annurev.psych.56.091103.070145 PMC3083636

[33] Bottera AR, Kambanis PE, De Young KP. The differential associations of shame and guilt with eating disorder behaviors. Eat Behav. 2020;39:101427. 32896681 10.1016/j.eatbeh.2020.101427

