# Supplementary material for: Validation of the Binge Eating Disorder Questionnaire (BED-Q) in Individuals with Type 2 Diabetes
Source: J Eat Disord. 2026 May 21;14:195. doi: 10.1186/s40337-026-01631-9 (PMC13495186; doi:10.1186/s40337-026-01631-9)
Supplement: Supplementary file 1 [file 40337_2026_1631_MOESM1_ESM.docx]

# Appendix 1: Binge Eating Disorder Questionnaire (BED-Q).

# (Danish version - validated).

Angiv, hvor mange episoder med tvangsoverspisning du har haft om ugen gennem de seneste tre måneder. Tvangsoverspisning betyder, at du inden for kort tid (fx to timer) spiser væsentligt mere, end hvad der er normalt, og at du ikke kan kontrollere, hvor meget du spiser.

Sæt kryds (“X”), ved hvor mange episoder med tvangsoverspisning du har haft om ugen.

Hvis det er forskelligt, hvor mange episoder med tvangsoverspisning du har haft om ugen gennem de seneste tre måneder, så angiv et gennemsnit (fx 20 episoder på 12 uger = 1-3 episoder om ugen).

Har du ikke haft episoder med tvangsoverspisning inden for de seneste tre måneder, skal du svare “Nej” i alle spørgsmålene.

Husk at besvare alle spørgsmålene (1 - 9).

| **Inden for de seneste tre måneder har du:** | **Nej** | **Ja, mindre end 1 gang  om ugen** | **Ja,**  **1-3 gange om ugen** | **Ja,**  **4-7 gange om ugen** | **Ja,**  **8-13 gange om ugen** | **Ja,**  **14 eller flere gange om ugen** |
| --- | --- | --- | --- | --- | --- | --- |
| 1. Haft episoder med tvangsoverspisning? (dvs. at du inden for et kort tidsrum (fx to timer) spiste væsentligt mere end hvad der er normalt) | 0 | 1 | 2 | 3 | 4 | 5 |
| 2. Mistet kontrollen over din spisning ved disse episoder? (fx at du ikke kunne stoppe med at spise eller kontrollere, hvor meget du spiste) | 0 | 1 | 2 | 3 | 4 | 5 |
| 3. Haft episoder med tvangsoverspisning, hvor du spiste hurtigere end normalt? | 0 | 1 | 2 | 3 | 4 | 5 |
| 4. Haft episoder med tvangsoverspisning, hvor du spiste, til du var ubehageligt mæt? | 0 | 1 | 2 | 3 | 4 | 5 |
| 5. Haft episoder med tvangsoverspisning, hvor du spiste store mængder mad, selvom du ikke var sulten? | 0 | 1 | 2 | 3 | 4 | 5 |
| 6. Haft episoder med tvangsoverspisning, hvor du spiste alene, fordi du var flov over, hvor meget du spiste? | 0 | 1 | 2 | 3 | 4 | 5 |
| 7. Haft episoder med tvangsoverspisning, hvor du følte væmmelse, depression eller skyld efter episoderne? | 0 | 1 | 2 | 3 | 4 | 5 |
| 8. Kastet op efter episoder med tvangsoverspisning? | 0 | 1 | 2 | 3 | 4 | 5 |

9. Er du meget belastet af dine episoder med tvangsoverspisning? (Ja/Nej)

# Scoringsnøgle ICD-11

## BED diagnostisk score (se gråfarvede felter i skemaet nedenfor)

For at opfylde kriterier for en BED diagnose skal:

- Både spørgsmål 1 og 2 være 2 eller højere.
- Spørgsmål 8 besvares med “Nej” eller “Ja, mindre end 1 gang om ugen”.
- Spørgsmål 9 besvares med “Ja”.

| **Indenfor de seneste tre måneder, har du:** | **Nej** | **Ja, mindre end 1 gang  om ugen** | **Ja,**  **1-3 gange om ugen** | **Ja,**  **4-7 gange om ugen** | **Ja,**  **8-13 gange om ugen** | **Ja,**  **14 eller flere gange om ugen** |
| --- | --- | --- | --- | --- | --- | --- |
| 1. Haft episoder med tvangsoverspisning? (dvs. at du inden for et kort tidsrum (fx to timer) spiste væsentligt mere end hvad der er normalt) | 0 | 1 | 2 | 3 | 4 | 5 |
| 2. Mistet kontrollen over din spisning ved disse episoder? (fx at du ikke kunne stoppe med at spise eller kontrollere, hvor meget du spiste) | 0 | 1 | 2 | 3 | 4 | 5 |
| 8. Kastet op efter episoder med tvangsoverspisning? | 0 | 1 | 2 | 3 | 4 | 5 |

9. Er du meget belastet af dine episoder med tvangsoverspisning? (Ja/Nej)

## BED belastningsscore:

Skalaen scores ved sammentælling af spørgsmål 1 og 2 (spørgsmål 8 og 9 tælles ikke med):

0 = Ingen BED symptomer

1-3 = Sub-kliniske BED symptomer

4-5 = Mulig indikation for BED, mild grad

6-7 = Mulig indikation for BED, moderat grad

8-9 = Mulig indikation for BED, svær grad

10-11 = Mulig indikation for BED, ekstrem grad

Minimum er 0; maksimum er 11

**Bemærk:** Den validerede diagnostiske cut-off (totalscore ≥ 12) gælder DSM-5-totalscoren (spørgsmål 1-7; 0-35; se Scoringsnøgle DSM-5 nedenfor). Der findes ingen valideret cut-off for ICD-11-belastningsscoren.

Følgende kriterier eller underkriterier fra ICD-11 er udeladt i BED-Q:

Essentielle (påkrævede) træk:

- Spørgsmål 2: *Kontroltab kan også forekomme som vanskeligheder ved at stoppe, når man begynder at spise, eller opgive at kontrollere spisningen, fordi man ved, man ikke vil lykkes*. Denne differentiering skønnes ikke at bidrage yderligere til patienternes forståelse, men kan introducere større bias eller forvirring hos patienterne, end den løser.
- Spørgsmål 8: Overspisningen er ikke ledsaget af upassende kompenserende adfærd*, der har til formål at forhindre vægtøgning*. Teksten i kursiv er udeladt, da differentieringen ikke skønnes at bidrage yderligere til patienternes forståelse, men kan introducere større bias eller forvirring hos patienterne, end den løser.
- *Symptomerne er ikke bedre forklaret som en anden medicinsk sygdom* (f.eks. Prader-Willi-syndrom) *eller psykisk lidelse* (f.eks. depression). Patienterne har ikke forudsætninger for at foretage en sådan skelnen.
- Der er markant belastning *eller forringelse i det personlige, familiemæssige, sociale, uddannelsesmæssige, jobmæssige eller andre vigtige områder af funktionsniveauet*. Teksten i kursiv er udeladt, da differentieringen ikke skønnes at bidrage yderligere til patienternes forståelse.

Yderligere kliniske træk:

- *Skelnen mellem subjektive og objektive overspisninger*. Mange BED-patienter skønnes ikke at have forudsætninger for at foretage en sådan skelnen.
- *Overspisningerne opleves som ”meget belastende” eller ”signifikant belastende”*. Mange BED-patienter skønnes ikke at have forudsætninger for at foretage en sådan skelnen.
- *BED hænger ikke sammen med overvægt*. BED-Q adresserer ikke overvægt.

Grænser til normalitet (tærskel):

- *Overspisning eller faste er ikke relateret til helligdage eller højtider*. Denne differentiering skønnes ikke at bidrage yderligere til patienternes forståelse, men kan introducere større bias eller forvirring.
- *Overspisning, der ikke opfylder kriterier for BED, bør ikke inkluderes*. Denne differentiering skønnes ikke at bidrage yderligere til patienternes forståelse, men kan introducere større bias eller forvirring.

# Scoringsnøgle DSM-5

## BED diagnostisk score (se gråfarvede felter i skemaet nedenfor)

For at opfylde kriterier for en BED diagnose skal:

- Både spørgsmål 1 og 2 være 2 eller højere.
- Mindst tre spørgsmål fra 3 til 7 være 2 eller højere.
- Spørgsmål 8 besvares med “Nej” eller “Ja, mindre end 1 gang om ugen”.
- Spørgsmål 9 besvares med “Ja”.

| **Indenfor de seneste tre måneder, har du:** | **Nej** | **Ja, mindre end 1 gang  om ugen** | **Ja,**  **1-3 gange om ugen** | **Ja,**  **4-7 gange om ugen** | **Ja,**  **8-13 gange om ugen** | **Ja,**  **14 eller flere gange om ugen** |
| --- | --- | --- | --- | --- | --- | --- |
| 1. Haft episoder med tvangsoverspisning? (dvs. at du inden for et kort tidsrum (fx to timer) spiste væsentligt mere end hvad der er normalt) | 0 | 1 | 2 | 3 | 4 | 5 |
| 2. Mistet kontrollen over din spisning ved disse episoder? (fx at du ikke kunne stoppe med at spise eller kontrollere, hvor meget du spiste) | 0 | 1 | 2 | 3 | 4 | 5 |
| 3. Haft episoder med tvangsoverspisning, hvor du spiste hurtigere end normalt? | 0 | 1 | 2 | 3 | 4 | 5 |
| 4. Haft episoder med tvangsoverspisning, hvor du spiste, til du var ubehageligt mæt? | 0 | 1 | 2 | 3 | 4 | 5 |
| 5. Haft episoder med tvangsoverspisning, hvor du spiste store mængder mad, selvom du ikke var sulten? | 0 | 1 | 2 | 3 | 4 | 5 |
| 6. Haft episoder med tvangsoverspisning, hvor du spiste alene, fordi du var flov over, hvor meget du spiste? | 0 | 1 | 2 | 3 | 4 | 5 |
| 7. Haft episoder med tvangsoverspisning, hvor du følte væmmelse, depression eller skyld efter episoderne? | 0 | 1 | 2 | 3 | 4 | 5 |
| 8. Kastet op efter episoder med tvangsoverspisning? | 0 | 1 | 2 | 3 | 4 | 5 |

9. Er du meget belastet af dine episoder med tvangsoverspisning? (Ja/Nej)

## BED belastningsscore:

Skalaen scores ved sammentælling af spørgsmål 1-7 fra 0-5 (spørgsmål 8 tælles ikke med):

0 = Ingen symptomer

1-9 = Sub-kliniske BED symptomer

10-14 = Mild BED (10-11 = under cut-off; 12-14 = sandsynlig BED)

15-21 = Moderat BED

22-28 = Svær BED

29-35 = Ekstremt svær BED

Minimum er 0; maksimum er 35

**Valideret diagnostisk cut-off:** En totalscore på 12 eller derover indikerer sandsynlig BED (AUC = 0.86; sensitivitet = 0.91; specificitet = 0.81; Youdens indeks = 0.71; PPV = 0.77; NPV = 0.92).

Belastningsscoren og den diagnostiske cut-off bruges til to forskellige formål: (1) belastningsscoren beskriver graden af BED-symptomer, og (2) cut-off’en (≥ 12) angiver, om der er sandsynlig BED.

En score på 10-11 betyder derfor milde BED-symptomer, men ikke sandsynlig BED. En score på 12 eller derover betyder sandsynlig BED; graden aflæses af belastningsscoren (12-14 = mild, 15-21 = moderat, 22-28 = svær, 29-35 = ekstremt svær).

Følgende kriterier eller underkriterier fra DSM 5 er udeladt i BED-Q:

Diagnostiske kriterier:

- Overspisningen er ikke ledsaget af upassende kompenserende adfærd*, som i bulimi, og forekommer ikke udelukkende i løbet af et sygdomsforløb med bulimi eller anoreksi*. Teksten i kursiv er udeladt, fordi mange BED-patienter ikke skønnes at have forudsætninger for at foretage en sådan skelnen.

Specificer, hvis:

- I delvis bedring
- I fuld bedring

Mange BED-patienter skønnes ikke at have forudsætninger for at foretage en sådan skelnen.

# Binge Eating Disorder Questionnaire (BED-Q).

# (English translation – not validated).

Please indicate how many binge eating episodes per week you have had during the past three months.

In a binge eating episode you eat a larger amount of food in a short period of time (e.g., within two hours) than what most people would eat under similar circumstances, and you lose control over your eating.

Check (“X”) the number of binge eating episodes you have had per week.

If the number of binge eating episodes per week has varied over the past three months , please indicate an average (e.g., 20 episodes in 12 weeks = 1-3 episodes per week).

If you have not had any binge eating episodes during the past three months, please check “No” to all questions.

Please make sure to answer all questions (1 - 9).

| During the past three months, have you: | No | Yes, less than once a week | Yes,  1-3 times per week | Yes,  4-7 times per week | Yes,  8-13 times per week | Yes,  14 or more times per week |
| --- | --- | --- | --- | --- | --- | --- |
| 1. Had binge eating episodes?  (i.e., within a short period of time (e.g., two hours) eaten a larger amount of food than what most people would)? |  |  |  |  |  |  |
| 2. Lost control over your eating during these episodes? (e.g., that you could not stop eating or control how much you were eating)? |  |  |  |  |  |  |
| 3. Had binge eating episodes, where you ate much more rapidly than normal? |  |  |  |  |  |  |
| 4. Had binge eating episodes where you ate until you were uncomfortably full? |  |  |  |  |  |  |
| 5. Had binge eating episodes where you ate large amounts of food, even though you were not hungry? |  |  |  |  |  |  |
| 6. Had binge eating episodes where you ate alone because you were embarrassed about how much you ate? |  |  |  |  |  |  |
| 7. Had binge eating episodes where you felt disgusted with yourself, depressed or guilty afterwards? |  |  |  |  |  |  |
| 8. Vomited after episodes of binge eating? |  |  |  |  |  |  |

9. Are you very distressed by the binge eating episodes? (Yes/No)

# BED diagnostic score (see gray cells in the table below)

- Both question 1 and 2 are 2 or higher.
- At least three of questions 3 to 7 are 2 or higher.
- Question 8 is “No” or “Yes, less than once a week”.
- Question 9 is “Yes”.

| During the past three months, have you: | No | Yes, less than once a week | Yes,  1-3 times per week | Yes,  4-7 times per week | Yes,  8-13 times per week | Yes,  14 or more times per week |
| --- | --- | --- | --- | --- | --- | --- |
| 1. Had binge eating episodes?  (i.e., within a short period of time (e.g., two hours) eaten a larger amount of food than what most people would)? | 0 | 1 | 2 | 3 | 4 | 5 |
| 2. Lost control over your eating during these episodes? (e.g., that you could not stop eating or control how much you were eating)? | 0 | 1 | 2 | 3 | 4 | 5 |
| 3. Had binge eating episodes, where you ate much more rapidly than normal? | 0 | 1 | 2 | 3 | 4 | 5 |
| 4. Had binge eating episodes where you ate until you were uncomfortably full? | 0 | 1 | 2 | 3 | 4 | 5 |
| 5. Had binge eating episodes where you ate large amounts of food, even though you were not hungry? | 0 | 1 | 2 | 3 | 4 | 5 |
| 6. Had binge eating episodes where you ate alone because you were embarrassed about how much you ate? | 0 | 1 | 2 | 3 | 4 | 5 |
| 7. Had binge eating episodes where you felt disgusted with yourself, depressed or guilty afterwards? | 0 | 1 | 2 | 3 | 4 | 5 |
| 8. Vomited after episodes of binge eating? | 0 | 1 | 2 | 3 | 4 | 5 |

9. Are you very distressed by the binge eating episodes? (Yes/No)

# BED severity score:

Calculate the total score for items 1-7 (0-5 per item). Question 8 is not included:

0 = No symptoms

1-9 = Subclinical symptoms of BED

10-14 = Mild BED (10-11 = below cut-off; 12-14 = probable BED)

15-21 = Moderate BED

22-28 = Severe BED

29-35 = Extremely severe BED

Minimum score = 0; maximum score = 35.

**Validated diagnostic cut-off:** A total score of 12 or higher indicates probable BED (AUC = 0.86; sensitivity = 0.91; specificity = 0.81; Youden’s index = 0.71; PPV = 0.77; NPV = 0.92).

The severity score and the diagnostic cut-off serve two different purposes: (1) the severity score reflects the severity of BED symptoms, and (2) the cut-off (≥ 12) indicates probable BED.

A score of 10-11 therefore indicates mild BED symptoms but not probable BED. A score of 12 or higher indicates probable BED; severity is classified according to the severity score (12-14 = mild, 15-21 = moderate, 22-28 = severe, 29-35 = extremely severe).
